# Comparison of linear mixed model analysis and genealogy-based haplotype clustering with a Bayesian approach for association mapping in a pedigreed population

**DOI:** 10.1186/1753-6561-6-S2-S4

**Published:** 2012-05-21

**Authors:** Golam R Dashab, Naveen K Kadri, Mohammad M Shariati, Goutam Sahana

**Affiliations:** 1 Department of Molecular Biology and Genetics, Faculty of Science and Technology, Aarhus University, DK-8830 Tjele, Denmark; 2 Department of Animal Science, Ferdowsi University of Mashhad, 91775 Mashhad, Iran

## Abstract

**Background:**

Despite many success stories of genome wide association studies (GWAS), challenges exist in QTL detection especially in datasets with many levels of relatedness. In this study we compared four methods of GWA on a dataset simulated for the 15^th ^QTL-MAS workshop. The four methods were 1) Mixed model analysis (MMA), 2) Random haplotype model (RHM), 3) Genealogy-based mixed model (GENMIX), and 4) Bayesian variable selection (BVS). The data consisted of phenotypes of 2000 animals from 20 sire families and were genotyped with 9990 SNPs on five chromosomes.

**Results:**

Out of the eight simulated QTL, these four methods MMA, RHM, GENMIX and BVS identified 6, 6, 8 and 7 QTL respectively and 4 QTL were common across the methods. GENMIX had the highest power to detect QTL however it also produced 4 false positives. BVS was the second best method in terms of power, detecting all QTL except the one on chromosome 5 with epistatic interaction. Two spurious associations were obtained across methods. Though all the methods considered the full pedigree in the analyses, it was not sufficient to avoid all the spurious associations arising due to family structure.

**Conclusions:**

Using several methods with divergent approaches for GWAS can be useful in gaining confidence on the QTL identified. In our comparison, GENMIX was found to be the best method in terms of power but it needs appropriate correction for multiple testing to avoid the false positives. This study shows that the issues of multiple testing and the relatedness among study samples need special attention in GWAS.

## Background

Despite many successes, genome-wide association studies (GWAS) still present major challenges. This is particularly true for samples drawn from a population with multiple levels of relatedness, such as population structure and/or familial relatedness. The efficiency of a GWAS method to detect a quantitative trait locus (QTL) depends on several factors, for example, the genetic architecture, allele frequency and heritability of the QTL, and the linkage disequilibrium with the marker. The population structure and relatedness of the samples may result in spurious associations. We applied a range of GWAS methods to map quantitative trait loci (QTL) in the simulated dataset provided by the 15th QTL-MAS workshop [1] and compared their efficiency in QTL detection with respected to this particular dataset.

We compared four different methods of GWAS, 1) Mixed model analysis (MMA); 2) Random haplotype model (RHM); 3) Genealogy-based mixed model (GENMIX) and 4) Bayesian variable selection method (BVS). The mixed model approach [2] utilizes the full relationship matrix and is the method of choice when the samples are drawn from a complex pedigreed population. The haplotype-based association methods using mixed models are generally regarded as more powerful than methods based on single markers [3,4] since they fully exploit LD information from multiple markers. On the other hand, genealogy based clustering of haplotypes in GENMIX not only consider the local LD but also takes the history of the origin of these haplotypes [5]. Contrary to the above three methods which analyze single markers or a few markers at a time, Bayesian variable selection [6] simultaneously fits multiple marker effects and avoids the problem of multiple testing. Therefore, it is useful to compare such Bayesian methods with more standard frequentist approaches where a single or a few SNPs are fitted at a time. The above-mentioned methods were compared for power, precision of location estimate, and type I error rate.

## Methods

The simulated population consisted of 20 sire families, each sire was mated to 10 dams and each full-sib family had 15 progeny. The phenotype was available for 10 progeny per full-sib family i.e. a total of 2000 individuals. There were five chromosomes each with 1998 SNPs at equal distance of 0.05 cM. The four GWAS method used for association mapping are described below.

### Mixed model analysis (MMA)

The association between each SNP and the phenotype was assessed by a linear mixed model analysis [2], using DMU software [7]. The model was as follows:

y=1μ+Xg+Zu+e

Where **y **is the vector of 2,000 phenotypes, **1 **is a vector of 1s of length 2,000, μ is the general mean, g is the additive effect of the SNP and **X **is a vector with genotypic indicators (0, 1, or 2) associating records to the marker effect, **u **is the random polygenic effect with the normal distribution $N\left(0,A\sigma_{u}^{2}\right)$, where **A **is the additive relationship matrix and $\sigma_{u}^{2}$ is the polygenic variance. **Z **is an incidence matrix relating phenotypes to the corresponding random polygenic effect, and **e **is a vector of random environmental deviates with the normal distribution $N\left(0,I\sigma_{e}^{2}\right)$, where $\sigma_{e}^{2}$ is the error variance and **I **is the identity matrix. Testing was done using a Wald test against a null hypothesis of *H*_0_:*g_i_*=0. The significance threshold was determined using a Bonferroni correction for the number of markers tested to obtain an experiment-wise P-value of 0.05.

### Random haplotype model (RHM)

The SNP genotype data were phased using software FastPhase [8].The haplotypes were 4 SNP long and they were tested for association sliding windows from SNP to SNP. The model for testing the association of the haplotypes at position j and the phenotype can be clarified in scalar form as follows:

$$y_{i}=\mu +u_{i}+q_{hm_{i}}+q_{hp_{i}}+e_{i}$$

Where *y_i _*is the phenotype of animal i, *μ *is the population mean, u_i _is the random polygenic effect, $q_{hm_{i}}$ and $q_{hp_{i}}$ are the random effects of the maternal and paternal haplotypes carried by individual i, and *e_i _*is the random residual effect as defined for MMA. The other random effect q was assumed to be normally distributed with mean zero and variances $I\sigma_{h}^{2}$ (assuming equal variance for paternal and maternal haplotypes). The significance of the haplotype substitution effect was assessed with a likelihood ratio test comparing the RHM model with a null-model containing mean, polygenic effect and random error terms but no haplotype effects. Analysis was performed using the DMU software package [7]. Significant threshold was fixed at genome wide 5% level after Bonferroni correction and the mid-point of significant haplotypes were considered as the putative QTL positions.

### Genealogy based mixed-model (GENMIX)

The efficiency of GENMIX for association mapping was described by Sahana et al. [5]. In contrast to regular genome-wide association studies where phenotypic differences are either associated with single markers or with groups of markers organized in to haplo-groups in a non-stratified fashion, here phenotypes were associated using a hierarchical approach. Both grouping of markers into haplo-groups and clustering of observed haplotypes was done based on local genealogies [9]. This method identifies the widest possible region surrounding a marker that allows construction of a genealogy forming a bifurcating tree without either recurrent mutation or recombination, in other words it satisfies the four-gamete condition of Hudson and Kaplan [10]. Each bifurcation in the binary tree corresponds to one bi-allelic marker. Splitting the tree at the top generates two clusters of haplotypes. Splitting the tree at any other node generates three clusters: one above the split point and two corresponding to the two branches below. For the analyses presented in this paper we split the tree at the top (one set of two clusters), the second level (two sets of three clusters) and at the third level (four sets of three clusters). Successively each clustering of haplotypes was included as a random effect in the model for analysis:

$$y_{i}=\mu +u_{i}+q_{h1_{i}}+q_{h2_{i}}+e_{i}$$

where *y_i _*is the phenotype of individual *i*, *μ *is the population mean, u_i _is as described above in the MMA; $q_{h1_{i}}$ and $q_{h2_{i}}$ are two haplotype effects of individual i, where *h1_i _*and *h2_i _*can take values 11, 12, 13, 22, 23, and 33 and $Var\left(q_{11},q_{12},q_{13},q_{22},q_{23},q_{33}\right)=I\sigma_{h}^{2}$ , $\sigma_{h}^{2}$ is the haplotype variance, and *e_i _*is a random residual as defined for MMA. The local genealogies were constructed using the software Blossoc (http://www.daimi.au.dk/~mailund/Blossoc/) and variance component analysis was carried out using the software DMU [7]. The significance of the SNP association was tested using likelihood ratio test and the significant threshold was fixed at genome-wide 5% level after Bonferroni correction for multiple testing for the total number of markers.

### Bayesian variable selection (BVS)

The method is based on specifying a mixture distribution for SNP effects while all SNP are fitted simultaneously in the model [6]. It was assumed that most markers had very small effects on the trait (98% of SNP in this analysis) and only few markers (2%) had large effects. The allocation of each SNP to either of these two distributions is done using an indicator variable in Gibbs sampling. The averaged mixture indicator estimates a posterior probability for that SNP to come from the distribution with large effects, which is interpreted as the probability for presence of an associated marker or QTL. The analysis was performed using BAYZ software [11] and the variances of the two mixture components were estimated. The SNP with posterior probability of the mixture indicator higher than 0.10; that corresponds to a Bayes factor of 5.5 were reported as QTL. In cases where adjacent markers showed a decreasing or increasing posterior probability of association due to linkage disequilibrium, only the SNP with highest probability was reported as QTL.

## Results

**Figure 1:**
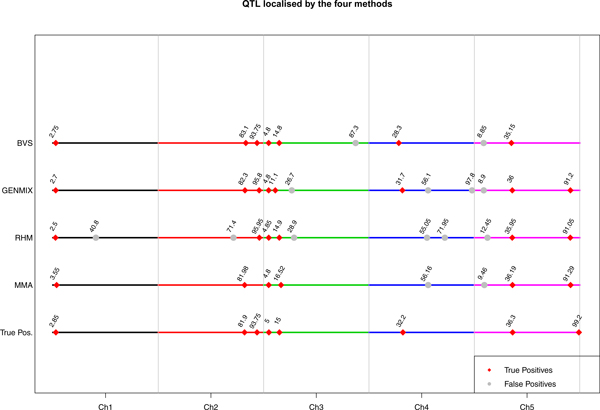
**Comparison of the positions of the simulated and detected QTL by four methods**. The positions of the detected QTL are given chromosome-wise for the four methods. The correct identifications are given in red diamonds and the false positives are given in gray circles.

The results of our analysis from four methods are summarised in Table 1 and graphically represented in Figure 1. A QTL was considered as identified if the putative location was within 10 cM of the true simulated location of the QTL. Out of the 8 simulated QTL, these four methods, MMA, RHM, GENMIX and BVS identified 6, 6, 8 and 7 QTL, respectively. Four QTL regions, one on chromosome 1 and 5 and two on chromosome 3, were identified by all the four methods. The numbers of false positives for these methods were 2, 6, 4 and 2 respectively (Table 1).

**Table 1:** Positions (cM) of identified QTL with the four methods

**Chr. No**.	True Position		Methods		
		
		MMA	RHM	GENMIX	BVS
1	2.85	3.55	2.50	2.70	2.75
2	81.90	81.90	*	82.30	83.10
2	93.75	*	95.95	95.80	93.75
3	5.00	4.80	4.85	4.80	4.80
3	15.00	16.52	14.90	11.10	14.80
4	32.20	*	*	31.70	28.30
5	36.30	36.19	35.95	36.00	35.15
5	99.20	91.29	91.05	91.20	*

False Positives		2	6	4	2

* False negatives

The effects of the QTL localised by MMA are given in table 2. The QTL with the biggest effect, explaining 10.2% of the variation in the phenotype was localised on chromosome 1 at 3.55 cM region. The 6 QTL detected by MMA together explained 18.4% of the phenotypic variance.

**Table 2:** QTL effects estimated by single marker analysis based on linear mixed model; fitting all the detected QTL simultaneously

**Chr. No**.	Position	Allele substitution effect¤	-log_10_(p-value)	Effect#
1	3.55	4.19	39.38	10.27
2	81.98	2.10	8.31	2.46
3	4.80	2.71	13.12	3.47
3	16.52	0.65	1.29	0.25
4	56.16	1.79	4.00	1.07
5	91.29	1.37	3.22	0.88

¤ Absolute value

#Percentage of phenotypic variance

Precision of the methods was assessed by the average of absolute differences between the positions of the simulated and the detected QTL, whenever it was identified. The QTL with the biggest effect on chromosome 1 was detected with high precision (on average ± 0.3 cM from the simulated QTL) and the epistatic QTL on chromosome 5 was detected with least precision (on an average 8 cM from the simulated QTL). In general the MMA identified the QTL with higher precision.

## Discussion

In our study we used additive models without considering the genetic architecture of the simulated QTL; however the methods performed well in localising the true simulated QTL. Out of the four methods employed, GENMIX performed comparatively better in QTL detection. It detected all the 8 simulated QTL and 6 were mapped accurately within a 2 cM region of the QTL region. However, it also identified 4 false positives (FP). The number of tests carried out in GENMIX was approximately 7 times the number of markers and we used Bonferroni multiple testing correction for the number of total marker but not for the total number of tests (i.e. ~7 times the number of markers) which could have resulted in increased number of false positives. Besides the number of haplotypes in a lineage goes down as we moved down the tree [5] which can give numerical instability. Out of these four FPs in GENMIX, two (on chromosome 4 and 5) were identified by other methods at the same location (Figure 1). Divergent approaches of GWAS picking up the same FP could be due to insufficient correction for family structure. A likely explanation is that some SNPs in these two regions were positively correlated (in linkage disequilibrium) with the QTL because of linkage (within family). It is thus not straightforward to distinguish true associations from spurious, regardless of the correction for the pedigree structure. This underlines the importance of replication study before a follow-up study can be taken up for identifying causal mutation underlying a QTL.

BVS was the second best method in terms of power to identify QTL and it had less FP compared to GENMIX. It detected all the simulated QTL except the one on chromosome 5 with epistatic interactions. BVS fits all the SNPs simultaneously and given that the first epistatic QTL was fitted in the model, there was a little chance for the second one to be significant in the model. In other words, the first QTL explains most of the variation induced by both QTL because of their dependency. Especially, this can happen if the epistasis is of additive by additive nature, where most of the epistatic variance is converted to additive [12]. In order to confirm this, we ran the MMA for all SNP on chromosome 5 where the first epistatic QTL was already in the model. As a result, the second epistatic QTL was not detected (results not shown).

The MMA identified six QTL. The two linked QTL on chromosome 2 were both identified by MMA but only the first one (the most significant) was reported in the workshop as the second QTL was not significant when fitted along with first one in the model. On the other hand RHM detected both of them but the first QTL was mapped 10 cM downstream the true QTL.

The highest significant SNP for the multi-allelic QTL on chromosome 1 (largest QTL) in MMA was 0.7 cM away from the true position, while the other methods mapped it closer to its position. No individual SNP (bi-allelic) can be in perfect LD with this QTL (multi-allelic) which might have resulted in poor precision for this QTL in MMA.

The imprinted QTL on chromosome 4 was only detected by GENMIX and BVS. The power of detection of the QTL will decrease if the model does not reflect the true genetic architecture of the QTL. However, GENMIX and BVS methods were sensitive enough to identify the imprinted QTL, though both of them model its effect as additive.

Sahana et al. [13] observed very high false positives when haplotypes were considered as fixed effects in the model. Because the frequency of some haplotypes can be very low, this could result in low accuracy of estimates and result in false positive when haplotypes are fitted as fixed effect. We expected this problem can be taken care by fitting haplotypes as random where the effects of the low frequent haplotypes will be regressed towards zero. However, RHM still had very high false positive rate.

## Conclusions

Using several methods in analysing GWA can be useful in gaining confidence on the QTL identified. Though, genealogy-based mixed model can be a powerful approach for GWAS, appropriate multiple testing correction is necessary to avoid false positives. Our study also shows that correction for pedigree relationship is not always enough to avoid spurious association arising due to family structure.

## Competing interests

The authors declare that they have no competing interests.

## Authors' contributions

All the authors have contributed in planning the study, analyses of data and writing the article.
